# Frequency distribution of health disorders in primary care—its consistency and meaning for diagnostics and nomenclature

**DOI:** 10.1007/s10354-024-01049-5

**Published:** 2024-07-22

**Authors:** Waltraud Fink, Otto Kasper, Gustav Kamenski, Sonja Zehetmayer, Dietmar Kleinbichler, Martin Konitzer

**Affiliations:** 1 Karl Landsteiner Institute for Systematics in General Practice, Straning 153, 3722 Straning, Austria; 2 Karl Landsteiner Institute for Systematics in General Practice, Reinöd 26, 3242 Texing, Austria; 3 Karl Landsteiner Institute for Systematics in General Practice, Ollersbachgasse 144, 2261 Angern/March, Austria; 4 Institute of Medical Statistics—Center for Medical Data Science, Medical University of Vienna, Spitalgasse 23, 1090 Vienna, Austria; 5 Karl Landsteiner Institute for Systematics in General Practice, Reiterhofgasse 1, 3385 Markersdorf, Austria; 6 Academic Teaching Practice, Hannover Medical School MHH, Hannover, Germany; 7 Karl Landsteiner Institute for Systematics in General Practice, Bahnhofstr. 5, 29690 Schwarmstedt, Germany

**Keywords:** Prevalence studies, Surveys in primary care, Nomenclature, Episode of care, Diagnostic technics and procedures, Prävalenz-Studien, Fälleverteilung in der Allgemeinmedizinpraxis, Nomenklatur, Behandlungsfall, Diagnostik

## Abstract

RN Braun observed that frequencies of health disorders in general practice are so consistent that he called his discovery “Case Distribution Law”. Our study compares morbidity data from methodologically similar surveys in primary care practices over a period of fifty years. Frequency ranks were determined for each observation period and the first 150 ranks were compared with Spearman’s correlation coefficients. All correlations were consistently positive. Frequency ranks were strikingly similar for surveys carried out at approximately the same time, especially when nomenclatural matching had been carried out before data collection. Ranks were also very similar where clear disease classifications were possible, but less so for non-specific symptoms.

The consistency of the distribution of health disorders helps develop diagnostic strategies (diagnostic protocols) and appropriate labeling for non-specific, diagnostically open symptom classifications. According to Braun’s considerations, the regularity of case distribution plays an important role in the professionalization of primary care.

**Supplementary Information:**

The online version of this article (10.1007/s10354-024-01049-5) contains supplementary material, which is available to authorized users.

## Abstract (de)

RN Braun beobachtete, dass Häufigkeiten von Gesundheitsstörungen in der Allgemeinmedizinpraxis so konsistent sind, dass er seine Entdeckung 1955 „Fälleverteilungsgesetz“ nannte. Unsere Studie vergleicht methodisch ähnliche Prävalenzerhebungen aus Hausarztpraxen innerhalb eines Zeitraums von fünfzig Jahren. Für jeden Beobachtungszeitraum wurden Häufigkeitsränge ermittelt und die jeweils ersten 150 Ränge mit den Korrelationskoeffizienten nach Spearman verglichen. Wir fanden insgesamt positive Korrelationen, die Häufigkeitsränge waren besonders ähnlich bei ungefähr zeitgleichen Erhebungen, und wenn vor der Datenerhebung eine nomenklatorische Abgleichung erfolgt war. Auch waren die Ränge sehr ähnlich, wo eindeutige Klassifizierungen möglich waren, hingegen weniger übereinstimmend bei unspezifischen Symptomen.

Die Konsistenz der Verteilung der Gesundheitsstörungen ermöglicht es, für die unspezifischen, diagnostisch offenen Symptomklassifizierungen diagnostische Strategien (Programmierte Diagnostik) und adäquate Bezeichnungen zu erarbeiten. Gemäß Brauns Überlegungen spielt damit die Regelmäßigkeit der Fälleverteilung eine wichtige Rolle bei der Professionalisierung der Allgemeinmedizin.

## Introduction

When primary care/family medicine began to see itself as a discipline in its own right, numerous morbidity studies were conducted. Frequencies were meant to show the burden of health disorders managed in primary care [1–3]. Medical educators needed information on the illnesses treated in general practices to establish training programs [4]. Some comparisons of different practices and different countries aimed to find common features for the definition of an international discipline of family medicine [5]. Other studies reflect on quality of care [6] or on coding [7].

Robert N Braun (1914–2007), Austrian general practitioner and researcher [8], also had an epidemiological approach in his research on primary health care. His goal was to develop a specific, diagnostic routine that would take into account the constraints and circumstances in general medical practices AND could be taught to newcomers to this field. He assumed that frequencies of health complaints seem to subconsciously influence and develop the diagnostic approach. Braun’s hypothesis agrees with cognitive associative memory models that suggest an influence of the experienced frequency of illness occurrence on diagnostic reasoning [9]. Further, he observed that experienced general practitioners are very good at estimating the frequency ranks of the diseases in their practices. Georga Cooke et al. confirm: “GPs develop an intuitive sense of the frequencies of the problems that they see” [10]. Braun inferred that there must be an underlying regularity in the occurrence of illnesses. In order to get reproducible results, he compared frequency data of the health problems which he encountered year by year: Frequency ranks were so similar that he published his discovery as “Fälleverteilungsgesetz”/“Case Distribution Law” [11]. As a book review puts it, “… *the author formulates what he calls the biological law of distribution of disease, the gist of which is that any group of people who live under similar conditions will react in a similar way to any factor relevant to health*.” [12]. Braun introduced the term “case statistics” for morbidity surveys in general medical practices in order to differentiate them from population based epidemiological surveys.

### Aim

Our objective is to reassess Braun’s finding of a consistency of frequencies by comparing case statistics from several practices as well as different observation periods. We hypothesized a significant correlation in frequency ranking between surveys, particularly between those that are close in time.

## Methods and material

Our material consists of temporally different datasets from five rural, physician-owned, solo primary care practices in Austria and one suburban practice in Switzerland, covering a span of more than five decades (1954–2012). In Austria (and similar in Switzerland in the eighties), patients have free access to a primary care practice of their choice; there is no patient list, but people tend to visit the nearest practice. All participating practices had a contract with the national obligatory health insurance, which pays doctors’ fees partly at a flat rate and partly on the service. The practices typically serve populations of 1000–2500 people of all ages. In order to see a specialist with a contract with the national health system, patients needed a referral letter. Since the introduction of the electronic patient card in 2005 this has been handled more loosely. However, experience shows that patients still tend to consult a family doctor due to long waiting times for an appointment in secondary care or because of further diagnostic or therapeutic needs [13].

Data were extracted from routine health disorder entries in medical records, independent of (electronic) billing records. The datasets of each practice only contained sickness records and no personal patient data. Braun describes a “case” as a health disorder that is assessed by the doctor during a patient visit; he also refers to it as “consultation result”. It is based on the smallest possible statistical rubric of health disorders which could be delimited and differentiated from each other. During one visit a doctor may have to deal with several cases of illness presented by the patient. All consultation results (= cases) were recorded episode-based by the physician personally. A change of the initial classification was rare. Chronic conditions were counted once annually. Non-sickness contacts, like vaccination or general health checks, were excluded. The denominator was the total number of illness cases per year in the respective practice. The rate per 1000 cases was calculated first annually and then averaged for the entire observation period (3, 5 or 10 years of observation) in each practice data set [14]. These case statistics had all been published before separately [15–20]. Additionally one practice data set with an observation period of a single year was included (Table 1).

**Table 1:** Overview of analyzed multiyear practice morbidity data: observation periods and number of recorded health problems (= consultation results, = illness episodes, = cases)

Period Practice	Observation period (month/year)	Duration (years)	Number of health problems	Ref
*Fifties*				
Braun1	10/54–09/59	5	**8146**^**a**^	[15]
*Seventies*				
Braun2	10/77–09/80	3	**7948**^**a**^	[16]
*Eighties*				
Landolt	07/83–06/88	5	**19,082**^**a**^	[17]
*Early nineties*				
Danninger	07/91–06/96	5	**17,255**	[18]
*Nineties*				
Fink1	10/89–09/99	10	**24,532**^**a**^	[19]
*New millennium*				
Fink2	2005–2009	5	**24,541**	[20]
Kasper	2005–2009	5	**32,605**	[20]
Kleinbichler	2012	1	**7502**	–

^a^Paper records

All others: electronic medical records

In order to make data sets from different practices comparable, we had to harmonize the nomenclature. From Braun’s early case statistics in primary care, up to 300 different diagnostic rubrics or entities had crystallized [21]. These are now defined and called *casugraphic* labels [22, 23]. Collaborating practice researchers, Danninger, Fink and Landolt had adopted the *casugraphic* nomenclature for their planned comparative analyses. The practitioners Kasper and Kleinbichler used their respective individual (practice adapted) nomenclature and coding (partly ICD-10, partly ICPC-2). The extracted lists of “diagnosis titles” (we will refer to them also as consultation results or episodes of care) were subsequently checked for obvious differences in nomenclature, i.e. in spelling and in granularity concerning especially detailed body regions. For rarer conditions that are not found in the *Casugraphy* the common medical nomenclature was used.

All malignancies, in order to have them better represented in the case statistics, were summarized into a single category. The same was done for fractures.

Frequency ranks were determined for every diagnostic rubric (diagnoses and symptom classifications were equivalent) in each practice. These rankings yielded the data sets for the comparative analysis of all practices at different times and locations. We performed a major analysis (A) comparing all different observation periods and a sub analysis (B) comparing single years from observation periods 1989–2012.

### Statistical analysis

A. Frequency ranks were computed for each of the eight observation periods and compared. The Spearman’s rank correlation coefficient was used as measure of the correlation of frequency ranks for each pairwise comparison. In case of tied ranks, average ranks were computed. Ties were especially common among the less frequent consultation results. Therefore the number of processed frequency ranks was limited to the 150 first ranking health problems. Beyond the 150th rank, health disorders become less frequent than one in 1000 cases.
  We can only provide simple Spearman correlations. We have information on the numbers of cases for different practices and years, however, we cannot attribute the cases to individual patients. A single patient can be included once or several times (for different or the same cases of illness). Thus it is not possible to distinguish *within*- and *between-group* variation. That is why no *p*-values were calculated for the correlation coefficients, as the assumption of independent observations would be violated.
B. The first ranking 150 illnesses recorded in the new millennium in the medical practices Fink and Kasper were our reference. All the ranks of the practices were compared pairwise on those 150 illnesses with Spearman’s correlation coefficients. At first health complaints in the data set of Fink2 were selected as basis of comparison. Then, for sensitivity analysis, the analyses were repeated with 150 most frequent consultation results in the same observation period in practice Kasper as comparator basis. Heatmaps of the results were generated.
C. Beyond that, we were interested how similar in ranking single years, in the same practice would be, compared to single years of other practices. Therefore an additional correlation analysis was carried out using annual data. These were available from four most recent practice surveys. Table 2 gives more details of these medical practices, such as the number of patients seen annually and how many different health problems were presented per patient on average. Again the Spearman’s rank correlation coefficient was used as measure of the correlation of frequency ranks for each pairwise comparison of the in total 21 individual practice years. However, the comparison ranking was determined here by summing up all cases in all practices over **all** years. The number of processed frequency ranks was also limited to the first 150 ranked health problems. All statistical analyses were performed using R 3.4.2 (https://cran.r-project.org/).

**Table 2:** Overview of analyzed yearly practice morbidity recordings

Practice	Observation period (month/year)	Number of patients	Number of health problems	Rate of health problems per person
**Fink1**				
Statistical year				
1	10/89–09/90	1178	2864	2.43
2	10/90–09/91	1129	2800	2.48
3	10/91–09/92	1068	2297	2.15
4	10/92–09/93	829^a^	2209	2.66
5	10/93–09/94	859^a^	2263	2.63
6	10/94–09/95	844^a^	1925	2.28
7	10/95–09/96	856^a^	2003	2.34
8	10/96–09/97	1154	2458	2.13
9	10/97–09/98	1226	2800	2.28
10	10/98–09/99	1207	2913	2.41
Total			**24,532**	
**Fink2**				
Statistical year				
1	2005	1538	4742	3.08
2	2006	1540	4776	3.10
3	2007	1644	5021	3.05
4	2007	1604	5023	3.13
5	2009	1525	4979	3.26
Total			**24,541**	
**Kasper**				
Statistical year				
1	2005	1751	6322	3.61
2	2006	1707	6409	3.75
3	2007	1754	6570	3.75
4	2007	1743	6475	3.71
5	2009	1744	6829	3.92
Total			**32,605**	
**Kleinbichler**	2012	2534	**7502**	2.96

^a^Patients treated on behalf of neighboring colleagues were not included

## Results

In the multiyear morbidity comparison, all primary care practices were positively correlated. Table 3 gives Spearman’s correlation coefficients based on the 150 most frequent Fink2 practice consultation results (**bold** numbers). The matrix shows that the practices correlated between 0.3 and 0.82. The overall very similar correlation coefficients, when Kasper’s first 150 ranks were used as reference, are shown in [brackets] (Table 3 and Supplementary_material_1_Heatmap_comparison_multiyear).

**Table 3:** Spearman’s correlation coefficients for 150 most frequent illness ranks in the multiyear observation periods

**Practices**		**New millennium**			**Nineties**		**Eighties**	**Seventies**	**Fifties**
		*Kleinbichler*	*Fink2*	*Kasper*	*Fink1*	*Danninger*	*Landolt*	*Braun2*	*Braun1*
**New millennium**	*Kleinbichler*	**1**	**–**	**–**	**–**	**–**	**–**	**–**	**–**
	*Fink2*	**0.56** [0.64]	**1**	**–**	**–**	**–**	**–**	**–**	**–**
	*Kasper*	**0.65** [0.67]	**0.67** [0.77]	**1**	**–**	**–**	**–**	**–**	**–**
**Nineties**	*Fink1*	**0.53** [0.56]	**0.67** [0.74]	**0.49** [0.63]	**1**	**–**	**–**	**–**	**–**
	*Danninger*	**0.48** [0.51]	**0.56** [0.63]	**0.47** [0.59]	**0.81** [0.82]	**1**	**–**	**–**	**–**
**Eighties**	*Landolt*	**0.49** [0.53]	**0.55** [0.58]	**0.4** [0.54]	**0.77** [0.79]	**0.76** [0.75]	**1**	**–**	**–**
**Seventies**	*Braun2*	**0.42** [0.45]	**0.47** [0.48]	**0.32** [0.44]	**0.75** [0.73]	**0.73** [0.75]	**0.78** [0. 79]	**1**	**–**
**Fifties**	*Braun1*	**0.38** [0.35]	**0.36** [0. 33]	**0.3** [0.34]	**0.73** [0. 65]	**0.65** [0.65]	**0.71** [0.72]	**0.77** [0.82]	**1**

Practice Fink2 as comparator—**bold figures**

Kasper’s 150 most frequent ranks as comparator—figures in [brackets]

The strongest correlation was found for practices with temporal proximity, where the same nomenclature (*Casugraphy*) was used prospectively (practices Landolt, Danninger and Fink1), whereas practices Fink2 and Kasper, measuring the same five years observation, correlated less closely, but still strongly (0.67—with practice Fink2 as comparator, and 0.77—with practice Kasper as comparator). The most recent practice data, but only from a single year, correlated weakly.

These 150 first ranks comprised at least 80% of the total number of illness episodes in all respective practices and observation periods, except for the very early survey of Braun, where the current first ranking illnesses represented only 71% of all his cases.

**Fig. 1:**
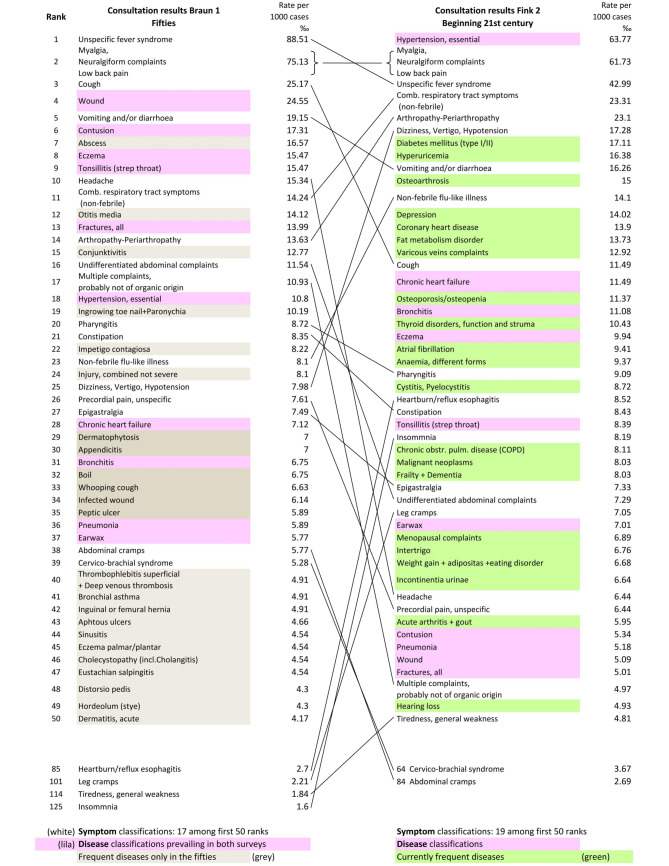
Observation periods Braun1 (1954–59) and Fink2 (2005–09) in comparison: First 50 ranks of consultation results in the respective practices; diseases are colored (*lila*—prevailing in the fifties and now, *green*—prevailing only nowadays, *beige*—only in the 1950s among the first 50 ranks), consultation results remaining undifferentiated symptom classifications (*not colored*) have arrows indicating increase or decrease over the years

Interesting differences in frequency ranks of illnesses fifty years apart are illustrated in Fig. 1. The fifty first ranking health disorders shown here represent 61.5% of all cases in Braun1 and 63.5% in Fink2, respectively. More than 20 diagnoses in the very early survey dropped out of the top ranking 50 health problems (especially pyogenic diseases like abscess, boils, paronychia, appendicitis, infected wounds etc.) and were “replaced” by diabetes, osteoarthrosis, osteoporosis, coronary heart disease, depression, atrial fibrillation, malignancies and others. But interestingly, half a century later almost the same top ranking **symptom** classifications can be found, indicated by the lines in Fig. 1. The percentage of cases with symptom classification among the 50 first ranking health problems was 33.3 in the fifties and 28.8 in 2005–2009. Details for all observation periods and up to 256 ranks of illnesses are provided as supplementary material (Supplementary_material_2_Table_256_ranks_english). 250 ranks are a number associated with a *regular frequency of occurrence *(Braun) of health disorders within a year in an average size primary care practice [16].

**Fig. 2:**
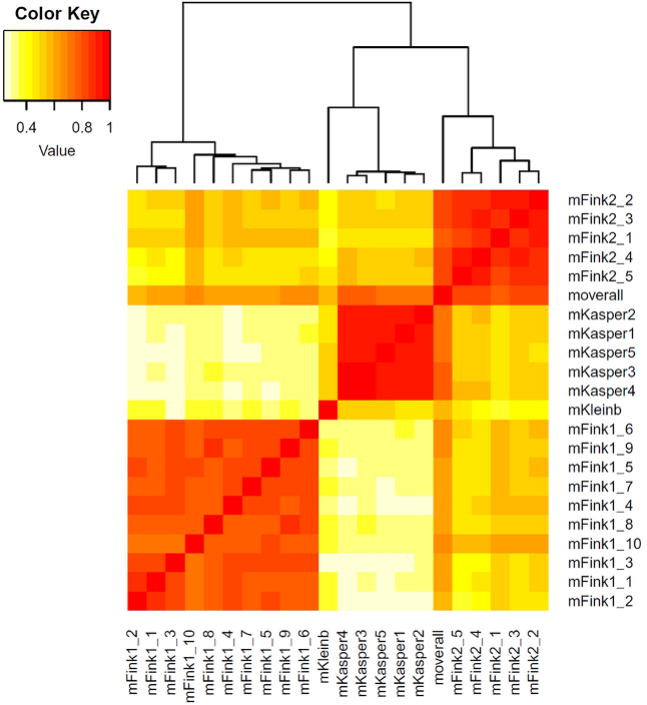
Heatmap—Visualization of correlation of 21 individual yearly rankings in the practices Fink (two observation periods), Kasper and Kleinbichler; as a comparison served a ranking, which was determined from the summed data (moverall); the better the correlation, the more intense the colors

The correlation analysis between the 21 individual annual recordings shows the strongest correlation—as expected—within the same practices (correlation coefficients in Fink1 0.71–0.85, Fink2 0.83–0.91 and Kasper 0.9–0.96). Between different practices the highest value reached was 0.57 (Supplementary_material_3_corOverallyearly_correlation_matrix). The heatmap visualizes the correlation (Fig. 2). Intense colors point to a high correlation.

**Fig. 3:**
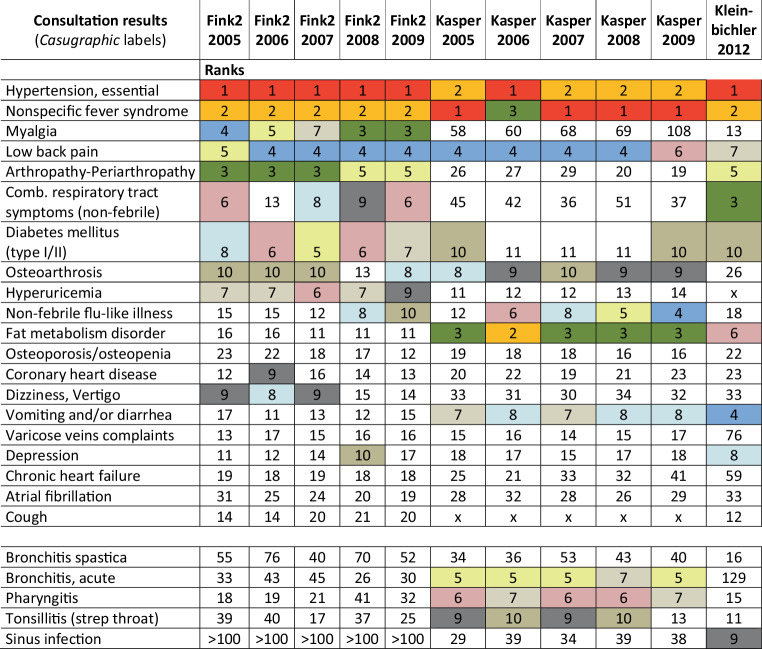
Comparison in three primary care practices (in the new millennium) of yearly rankings of 20 consultation result; (the ranks 1–10 are *colored*; in the lines of the health problem (listed according Fink2 in 2009) the respective ranks in each year and in each of the three practices are shown)

Figure 3 lists the most frequent illnesses by year and respective ranks. It shows that, within the same practice, ranks hardly differ from one year to the next.

**Fig. 4:**
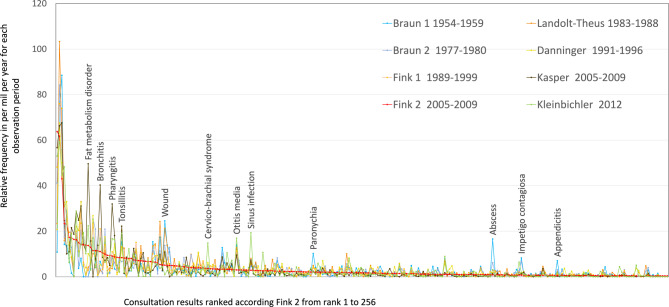
256 frequency ranks of consultation results (ranked according practice Fink2). A graphic comparison for all practices of all multiyear observation periods; interesting “peaks” as described in the results are highlighted; detailed figures in Supplementary_material_2_Table_265_ranks_english

Additional knowledge may be drawn from the cognition, that the overall distribution of illness frequencies can be described as an example of the 20/80 power-law (Pareto) (Fig. 4). It illustrates that the majority of cases is found in the first ranking 20% of the average of 300–400 ranks of different health problems. This is true in all decades.

## Discussion

### Law of regular frequency distribution

Braun first started with a visual presentation of his year-over-year frequency data and then compared them with data from the UK [11]. There was an obvious similarity as there was in our material (Fig. 4). Early morbidity surveys in Canada by Lynn Curry and Karen MacIntyre also described “*similarity between the morbidity patterns in all areas*”. They already used a statistical method for their comparison: Frequency ranks in 15 profile studies in family practice showed similarity with Kendall’s coefficient of concordance of 0.65 [4]. Later Braun and Haber used Spearman’s correlation analysis and had coefficients of 0.47–0.71 [24]. It again confirmed the similarity. Our study found positive Spearman’s correlation coefficients between 0.30 and 0.82 when all different practices were compared (Table 3 correlation matrix). The results support our hypothesis of a general inherent similarity. If variations occur they are due, on the one hand, to the different survey periods, on the other hand, to differences in nomenclature.

### Influence of different observation periods

Braun himself observed that the ranking was influenced by changing living conditions [25]. Our comparison over five decades equally reflects these changes. We see the well-known epidemiologic changes in diseases of affluence like diabetes and hypertension. Likewise, life expectancy has risen, which also influences the types of disorders that are presented to primary care practitioners. Furthermore, the increase of recorded coronary heart disease, osteoporosis, atrial fibrillation or depression may be due to better diagnostic procedures and therapeutic options. The high figures of hyperuricemia, of lipid or thyroid disorders in all recent surveys must not only be interpreted as a change in the morbidity spectrum, but could be the result of lowered thresholds, or more frequent testing or better monitoring of chronic conditions in electronic medical records (Supplementary_material_2_Table_256_ranks_english).

Changes within the healthcare system may play a role in the case distribution of health disorders seen by primary care physicians. During our observation period, the rise of private medicine had only just begun, so there was no noticeable impact. From Prosenc’s observation we know that an increase in the number of practicing specialists in his town, in the sixties, led to an overall decrease in cases in his primary care practice [26]. However, in contrast to Prosenc’s findings Fink’s practice—comparing observation periods in the nineties with 2005–2009—showed an increase in both patients and the number of health problems per patient (Table 2). A second point of consideration is that out-of-hours services in our hospital outpatient departments have increased in recent years. Patients may or may not be sent back to the practitioner. No doubt, it would be useful to monitor practice morbidity in sentinel practices, in different medical fields and institutions, and to assess the influence of changes in the healthcare system on the case distribution.

### Nomenclature as the pivotal point

Based on the expected consistency of illness frequencies especially between surveys close in time, we looked for the relationship between the labeling of health disorders and the similarity of case frequency distributions.

The rankings of illnesses in the different practices in the eighties and nineties, where primarily *casugraphic* labels were used, are highly consistent (up to 0.82), whereas the most recent data, where the physicians’ labels were harmonized retrospectively, showed less strong correlations with each other (Table 3). But, as visualized in the heatmap (Fig. 2), the comparison of consultation results year by year within the same practices yielded a very strong correlation with a Spearman correlation coefficient of up to 0.96 (Supplementary_material_3_corOverallyearly and Fig. 4). Similarly, Crombie et al. had observed a “*consistency of any individual doctor’s pattern of diagnostic recording from one year to another*” [27]. A closer look at the respective first 10–20 ranks in our yearly comparison reveals a possible source of disparities (Fig. 3): In the process of harmonizing the nomenclature for the analyses, identical clinical expressions were analyzed as they were recorded. As shown in Fig. 3, we found that some ranks differ considerably among practitioners but are very consistent every year within the same practice (e.g. myalgia, acute bronchitis, cough, strep throat, dizziness). These results suggest that clinical terms were used with a variable individual meaning. In the transnational study by Jean Karl Soler and collaborators, using International Classification of Primary care (ICPC-2) codes, the **reasons** for encounter (RfE) codes showed, “*striking similarities in the incidence or prevalence rates*”, but considerable variability in the **consultation results**, coded as “episode of care” (EoC) [5]. As seen in many studies, similar ranks are observed especially when clear medical terms are available or when clinical conditions are evident (e.g. excessive earwax, certain skin diseases) [3, 5, 28]. The better a clinical condition can be assessed by general practitioners, e.g. hypertension, diabetes, low back pain, the more consistency is found with other practices. When dealing with nonspecific symptoms however, physicians develop their own specific ways of assessing conditions. They seem to have different preferred labels and different codes when no firm diagnosis is reached [7]. There have been several attempts to subsume primary care doctors’ colloquial clinical terms under appropriate codes that are ideally compatible with ICD-codes. It started in the sixties with the Royal College of General Practitioners’ Classification, the US Ambulatory Medical Care Classification of Symptoms (NAMCS), and then early versions of the ICPC, the International Classification of Health Problems of Primary care (ICHPPC) [29]. This was followed by Oscar Rosowsky’s efforts to integrate *Casugraphic* labels into ICD, as well as to assist practitioners in their diagnostic considerations [30].

When there is variance in morbidity figures, it seems possible that there is variance in the diagnostic approach too [31]. Braun described his research experience: “*The entries must be made more precisely, without violating the facts. For this purpose, it has been found to be of particular importance to delimit each individual case or each individual rubric from all practically important diagnostically related cases and rubrics. … The way to a usable classification is inextricably linked to a realistically developed and sophisticated practical diagnostics and vice versa*. *Diagnostics and classification represent a unit, like columns of fluid in communicating vessels*” (Braun) [11, p. 60, 61^1^]. Diagnostic reasoning influences classification and vice versa [32].

Beyond epidemiological issues, the case distribution provides insights into the everyday challenges of a primary care physician. The comprehensive approach taught in medical school often cannot be followed due to known constraints and limitations in frontline medical care. “*A very high percentage of cases … will not be diagnosed in the accepted sense of the word, but can only be classified according to the leading symptom or symptom complexes. Many of these will be minor illnesses, clearing up after a short course. … Yet among this mass of clinical material there will occur, rarely, but regularly, potentially dangerous conditions*” [33]. The power law morphology of illness ranking reminds that diagnostic considerations have to do with risk management. In the so called “fat tail” the least certain diagnostic units prevail, like fever, respiratory symptoms, pain (muscular-skeletal, abdominal or precordial), headache, dizziness (Fig. 4). Herein lies a high risk of a hidden life threatening condition or “black swan” event, as they are called by today’s scientific forecasting [34]. The physician must always be prepared for such a rare event, despite its low probability. An unwarranted disease label here is a risk for premature closure [35–37]. As diagnoses are traditionally expected, physicians may hesitate to classify on the symptom level [38]:

This again leads to nomenclature issues. “… *new codes to denote diagnostic uncertainty in the patient-provider encounter*” [39] are needed. Knowledge of the “law of case distribution” suggests that we can expect a manageable number of 200–300 health problems without a confirmed diagnosis to occur regularly. The casugraphic diagnostic rubrics emerged as a by-product of Braun’s case statistic [40]. They were defined by delimiting each rubric from related rubrics and by listing the most important potentially dangerous conditions to be considered in the differential diagnostic procedure [23, 41].

It is conceivable to integrate these casugraphic units as prototypes of consultation results into automatic coding software. Kazem Sadegh-Zadeh, author of the “Handbook of Analytic Philosophy of Medicine” [42] considered Braun’s approach to be more or less the only approach suitable for unambiguous and automatic data assignment (M. Konitzer, personal communication, 2012). Embedded in a problem-oriented electronic patient file, already being developed by Wolfgang Edinger, it could be a helpful tool both for the diagnostic work with the patient and for coding [43, 44]. Undifferentiated symptoms and syndromes become as manageable as if they were diagnoses, but with the reminder of an open situation, where attentive observation is required.

## Conclusion

The typical and consistent frequency distribution of health disorders enables the case statistics to be used as a basic tool to arrive at unambiguous rubrics for further developing diagnostic strategies and protocols appropriate for primary care. Common terminology among physicians and a uniform diagnostic approach will lead to more comparable data in future research, and, last but not least, will increase patients’ safety.

### Strength and limitations

Data comes from everyday practice and was collected very conscientiously and carefully by physicians, covering the entire spectrum of diseases and an extremely long period of time. A limitation is that we had to rely on relatively old published data when electronic medical records did not exist. The fact that the recorded diagnoses were not influenced by financial incentives, as observed in current health insurance cost analyses [45, 46], can be seen as a strength.

We used the *casugraphic* nomenclature which was most commonly employed in our surveys, as subsequent transcoding into ICD or ICPC would have resulted in inaccuracies and a significant loss of detailed information.

General practices were not sampled at random, but all of them operated within an unselected population and within the national health care system. The patient structure is only partially known or was not recorded because the survey was limited to recording health disorders. A population-based survey with precise age and gender distribution was not intended; the focus was on the physician’s recording of the episode of illness he or she was consulted for.

## Supplementary Information

Supplementary_material_1_Heatmap_comparison_multiyear. Visualization of correlation of multiyear observation periods in six different practices

Supplementary_material_2_Table_256_ranks_english. Detailed figures of all multi-years practice surveys: 256 ranks of consultation results/ICD-10/ICPC^2^-codes/type of classification/rank/total number/rate per 1000 cases

Supplementary_material_3_corOverallyearly_correlation_matrix. Spearman correlation coefficient matrix for 21 individual statistical years

## Footnotes

1 “Die Eintragungen mußten genauer, und ohne den Tatsachen Gewalt anzutun, erfolgen. Zu diesem Behufe erwies es sich ganz besonders wichtig, jeden einzelnen Fall bzw. jede einzelne Rubrik gegen alle praktisch wichtigen, diagnostisch benachbarten Fälle und Rubriken hin abzugrenzen” [11, S. 60]. “Der Weg zu einer brauchbaren Rubrizierung ist untrennbar verknüpft mit der Entwicklung einer realistisch ausgefeilten, praktischen Diagnostik und umgekehrt. Diagnostik und Rubrizierung stellen eine Einheit dar, wie Flüssigkeitssäulen in kommunizierenden Gefäßen” [11, S. 61].
