# Supplementary figures and images for: Frequency distribution of health disorders in primary care—its consistency and meaning for diagnostics and nomenclature
Source: Wien Med Wochenschr. 2024 Jul 22;175(5-6):99–109. doi: 10.1007/s10354-024-01049-5 (PMC11928369; doi:10.1007/s10354-024-01049-5)

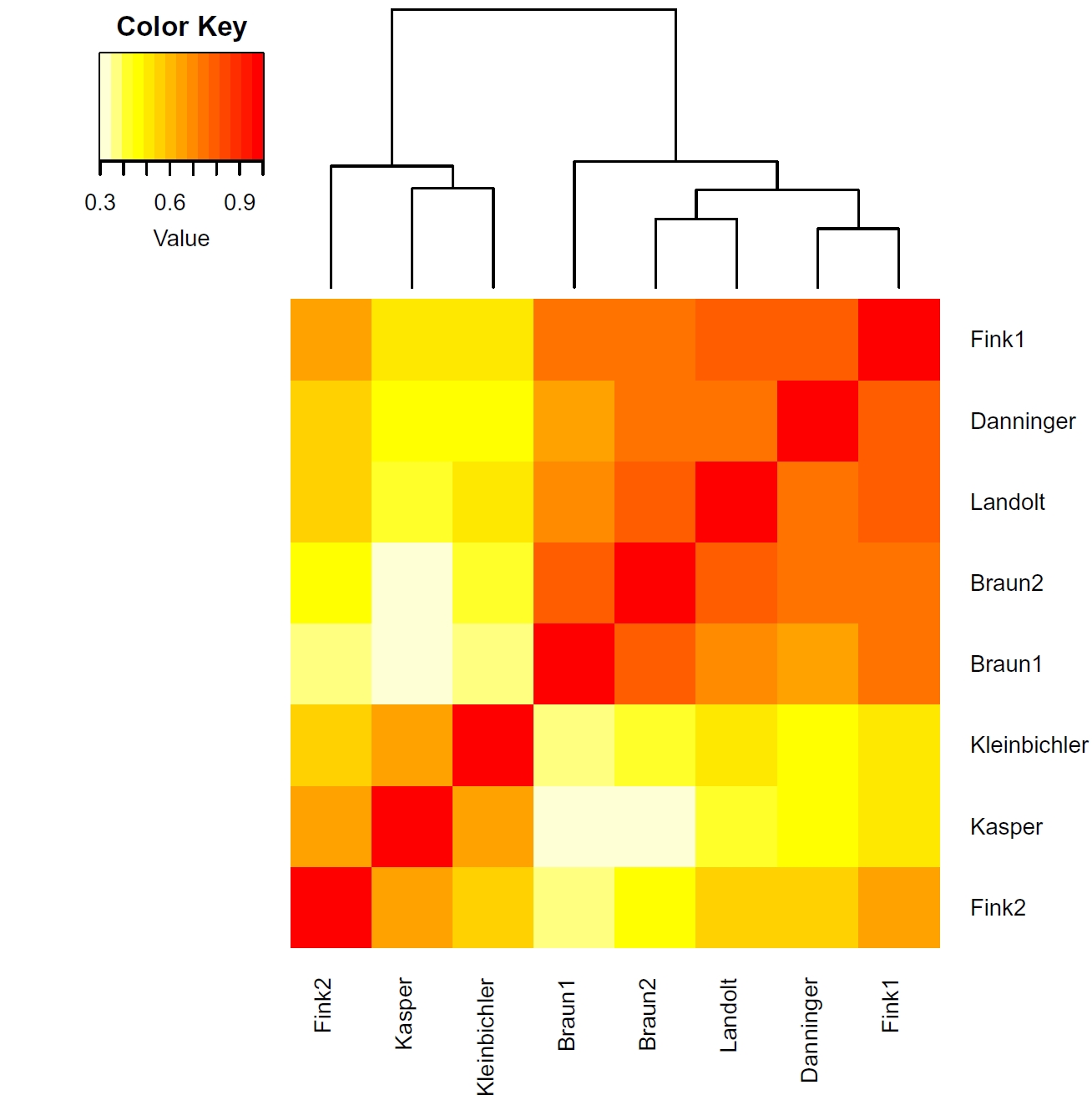

Supplement: Supplementary file 1 — Supplementary_material_1_Heatmap_comparison_multiyear. Visualization of correlation of multiyear observation periods in six different practices [file 10354_2024_1049_MOESM1_ESM.jpg]
