# Supplementary material for: Frequency distribution of health disorders in primary care—its consistency and meaning for diagnostics and nomenclature
Source: Wien Med Wochenschr. 2024 Jul 22;175(5-6):99–109. doi: 10.1007/s10354-024-01049-5 (PMC11928369; doi:10.1007/s10354-024-01049-5)
Supplement: Supplementary file 2 — Supplementary_material_2_Table_256_ranks_english. Detailed figures of all multi-years practice surveys: 256 ranks of consultation results/ICD-10/ICPC2-codes/type of classification/rank/total number/rate per 1000 cases [file 10354_2024_1049_MOESM2_ESM.pdf]

Rank Fink2	Results of consultation	ICD 10 codes	ICPC 2 codes	Type of classification: A=symptom, B=group of symptoms	C=image of a disease or D=diagnosis	Kleinbichler 2012 n = 7502 Rank	n	Rate per 1000 cases (‰)	Fink2 2005-09 n = 24.541 Rank	n	Rate per 1000 cases (‰)	Kasper 2005-09 n = 32.605 Rank	n	Rate per 1000 cases (‰)	Fink1 1989-99 n = 24.532 Rank	n	Rate per 1000 cases (‰)	Danninger 1991-96 n = 17.255 Rank	n	Rate per 1000 cases (‰)	Landolt-Theus 1983-88 n = 19.082 Rank	n	Rate per 1000 cases (‰)	Braun2 1977-80 n = 7.948 Rank	n	Rate per 1000 cases (‰)	Braun1 1954-59 n = 8.146 Rank	n	Rate per 1000 cases (‰)
1	Hypertension, essential	I10	K86		C	2	397	52.92	1	1565	63.77	3	1848	56.68	3	1181	48.14	8	358	20.75	6	445	23.32	3	327	41.14	18	88	10.80
2	Myalgia, Neuralgiform complaints Low back pain	M79.1,M79.2 M54.2/5/9 M53.3	L01,02,L04,05, L18,19 A01 L03	A/B		1	445	59.32	2	1515	61.73	2	2161	66.28	1	1865	76.02	1	1165	67.52	1	1971	103.29	1	669	84.17	2	612	75.13
3	Nonspecific fever syndrome	R68.8	A03	B		4	335	44.65	3	1055	42.99	1	2204	67.60	2	1813	73.90	2	864	50.07	2	1121	58.75	2	443	55.74	1	721	88.51
4	Comb. respiratory tract symptoms (non-febrile)	J06.9	R74	B		3	362	48.25	4	572	23.31	10	804	24.66	4	802	32.69	5	532	30.83	10	302	15.83	9	139	17.49	11	116	14.24
5	Arthropathy-Periarthropathy	M25.9	L07,08,10,11,12, L13,15,16,20	B		6	174	23.19	5	567	23.10	23	328	10.06	5	624	25.44	3	570	33.03	4	530	27.77	4	232	29.19	14	111	13.63
6	Dizziness, Vertigo, Hypotension	R42,I95.9	N17,H82	A		36	47	6.26	6	424	17.28	20	357	10.95	11	479	19.53	12	241	13.97	9	315	16.51	11	131	16.48	25	65	7.98
7	Diabetes mellitus (type I/II)	E10,E11	T89,T90		D	11	141	18.79	7	420	17.11	12	703	21.56	12	469	19.12	24	151	8.75	28	161	8.44	13	102	12.83	123	13	1.60
8	Hyperuricemia	E79.0	A91,T99		D	0	0	0.00	8	402	16.38	13	609	18.68	81	70	2.85	44	87	5.04	205	11	0.58	242	2	0.25	249	1	0.12
9	Vomiting and/or diarrhoea	A09,K52.9	D10,D11,D73	A/B		5	216	28.79	9	399	16.26	8	939	28.80	10	490	19.97	7	382	22.14	8	355	18.60	10	137	17.24	5	156	19.15
10	Osteoarthrosis	M17.9,16.9,19.9	L89,90,91,92		D	25	68	9.06	10	368	15.00	9	810	24.84	17	278	11.33	9	351	20.34	19	210	11.01	48	36	4.53	64	30	3.68
11	Non-febrile flu-like illness	R53,R68.2,Z03.9	A05	B		18	107	14.26	11	346	14.10	7	1013	31.07	6	578	23.56	4	566	32.80	3	629	32.96	8	163	20.51	23	66	8.10
12	Depression	F32,F33	P76		C	8	145	19.33	12	344	14.02	16	466	14.29	29	156	6.36	43	87	5.04	39	107	5.61	51	36	4.53	0	0	0.00
13	Coronary heart disease	I25	K74		D	24	76	10.13	13	341	13.90	19	368	11.29	58	97	3.95	167	15	0.87	326	0	0.00	0	0	0.00	0	0	0.00
14	Fat metabolism disorder	E78	T93		D	7	168	22.39	14	337	13.73	4	1617	49.59	78	73	2.98	18	175	10.14	53	79	4.14	0	0	0.00	0	0	0.00
15	Varicous veins complaints	I83.9	K95		D	68	22	2.93	15	317	12.92	15	512	15.70	28	163	6.64	25	142	8.23	38	111	5.82	24	67	8.43	65	29	3.56
16	Cough	R05	R05	A		12	128	17.06	16	282	11.49	0	0	0.00	7	571	23.28	6	463	26.83	22	196	10.27	5	203	25.54	3	205	25.17
17	Chronic heart failure	I50.9	K77		C/D	56	30	4.00	17	282	11.49	32	253	7.76	18	276	11.25	19	162	9.39	26	173	9.07	7	166	20.89	28	58	7.12
18	Osteoporosis/osteopenia	M80,M81.9	L95		D	20	82	10.93	18	279	11.37	17	451	13.83	165	23	0.94	189	11	0.64	281	2	0.10	0	0	0.00	0	0	0.00
19	Bronchitis	J20	R78		C	13	127	16.93	19	272	11.08	5	1313	40.27	8	516	21.03	40	92	5.33	78	54	2.83	62	29	3.65	31	55	6.75
20	Thyroid disorders, function and struma	E00/01/03/05, E89.0/ E04	T85,T86 T81		D	26	68	9.06	20	256	10.43	28	278	8.53	74	79	3.22	55	76	4.40	127	31	1.62	149	11	1.38	63	31	3.81
21	Eczema	L30.9,L20	S87		C	37	47	6.26	21	244	9.94	34	230	7.05	14	342	13.94	10	302	17.50	18	222	11.63	6	166	20.89	8	126	15.47
22	Atrial fibrillation	I48	K78	A or	D	32	54	7.20	22	231	9.41	30	273	8.37	47	107	4.36	232	3	0.17	85	50	2.62	137	13	1.64	142	11	1.35
23	Anaemia, different forms	D50.9,D51,D64.9	B80,81,82	A or	D	27	67	8.93	23	230	9.37	41	161	4.94	89	64	2.61	79	55	3.19	59	72	3.77	146	11	1.38	108	16	1.96
24	Pharyngitis	J02.9	R74	A		15	125	16.66	24	223	9.09	6	1042	31.96	19	269	10.97	45	85	4.93	63	68	3.56	21	72	9.06	20	71	8.72
25	Cystitis, Pyelocystitis	N30.9	U71		C/D	16	123	16.40	25	214	8.72	14	590	18.10	23	210	8.56	11	250	14.49	25	184	9.64	35	49	6.17	66	28	3.44
26	Heartburn/reflux esophagitis	R12	D03	A		49	37	4.93	26	209	8.52	40	163	5.00	114	45	1.83	158	17	0.99	70	62	3.25	73	25	3.15	85	22	2.70
27	Constipation	K59.0	D12	A		91	15	2.00	27	207	8.43	75	68	2.09	30	155	6.32	74	62	3.59	54	78	4.09	27	62	7.80	21	68	8.35
28	Tonsillitis (strep throat)	J03.9,J03.0,J03.8	R76		C	10	143	19.06	28	206	8.39	11	724	22.21	9	500	20.38	13	228	13.21	15	244	12.79	33	53	6.67	9	126	15.47
29	Insomnnia	F51.0,G47.0,G47.9	P06	A		93	15	2.00	29	201	8.19	104	47	1.44	57	98	3.99	87	49	2.84	17	239	12.52	29	58	7.30	125	13	1.60
30	Chronic obstr. pulm. disease (COPD)	J44	R95		C	40	43	5.73	30	199	8.11	27	279	8.56	190	14	0.57	302	0	0.00	343	0	0.00	0	0	0.00	350	0	0.00
31	Malignant neoplasms	D00-D09,D37-D48, C00-C97	Respective chapter		D	19	92	12.26	31	197	8.03	61	90	2.76	25	182	7.42	73	62	3.59	74	57	2.99	75	25	3.15	92	20	2.46
32	Frailty + Dementia	F00,F01,F03,R54	P70,P05		C	72	20	2.67	32	197	8.03	43	151	4.63	40	119	4.85	57	75	4.35	40	103	5.40	45	38	4.78	89	21	2.58
33	Epigastralgia	R10.1	D02	A		23	76	10.13	33	180	7.33	18	404	12.39	32	143	5.83	23	156	9.04	27	164	8.59	56	30	3.77	27	61	7.49
34	Undifferentiated abdominal complaints	R10.4,R19,K58	D06,D25,D28,29,D 93	B		29	62	8.26	34	179	7.29	38	177	5.43	13	370	15.08	14	210	12.17	20	210	11.01	19	79	9.94	16	94	11.54
35	Leg cramps	R25.2	L19	A		242	1	0.13	35	173	7.05	194	13	0.40	62	95	3.87	92	45	2.61	79	54	2.83	127	14	1.76	101	18	2.21
36	Earwax	H61.2	H81		D	50	35	4.67	36	172	7.01	31	263	8.07	20	260	10.60	22	159	9.21	13	258	13.52	32	55	6.92	37	47	5.77
37	Menopausal complaints	N95.1	X11	B		232	1	0.13	37	169	6.89	22	344	10.55	54	102	4.16	39	93	5.39	64	67	3.51	23	69	8.68	88	21	2.58
38	Intertrigo	L30.4	S88		C/D	234	1	0.13	38	166	6.76	85	60	1.84	106	51	2.08	100	39	2.26	183	15	0.79	163	10	1.26	156	9	1.10
39	Weight gain + adipositas + eating disorder	R63.5,E66	T82,83	A or	C/D	31	55	7.33	39	164	6.68	95	52	1.59	83	69	2.81	61	73	4.23	29	158	8.28	43	40	5.03	116	14	1.72
40	Incontinentia urinae	N39.3,N39.4	U04	A		233	1	0.13	40	163	6.64	76	68	2.09	95	60	2.45	111	33	1.91	120	33	1.73	202	5	0.63	221	3	0.37
41	Headache	R51,G44.2	N89	A		34	52	6.93	41	158	6.44	57	93	2.85	21	246	10.03	27	138	8.00	14	257	13.47	15	92	11.58	10	125	15.34
42	Precordial pain, unspecific	R07.2	K01,02,K24	A		43	42	5.60	42	158	6.44	42	151	4.63	15	338	13.78	21	159	9.21	11	274	14.36	22	69	8.68	26	62	7.61
43	Acute arthritis + gout	M13.9,M10.9	L91,T90		C	42	42	5.60	43	146	5.95	37	181	5.55	31	151	6.16	20	161	9.33	41	101	5.29	158	10	1.26	83	23	2.82
44	Contusion	S00-S90, T14.0	S16		C	59	28	3.73	44	131	5.34	24	321	9.85	22	228	9.29	28	132	7.65	5	462	24.21	16	90	11.32	6	141	17.31
45	Pneumonia	J18.9	R81		C/D	52	33	4.40	45	127	5.18	58	93	2.85	33	142	5.79	42	90	5.22	55	77	4.04	101	19	2.39	36	48	5.89
46	Wound	T14.1	S18		D	143	7	0.93	46	125	5.09	21	352	10.80	27	166	6.77	15	194	11.24	7	406	21.28	12	109	13.71	4	200	24.55
47	Fractures, all	S00-etc.	L72,L74,L76		D	73	20	2.67	47	123	5.01	44	144	4.42	50	105	4.28	38	98	5.68	12	261	13.68	28	59	7.42	13	114	13.99
48	Multiple complaints, probably not of organic origin	F44,45	P75	B		106	13	1.73	48	122	4.97	0	0	0.00	34	140	5.71	83	51	2.96	99	40	2.10	14	102	12.83	17	89	10.93
49	Hearing loss	Z73.6,H91.1	H28,H84	A or	D	174	4	0.53	49	121	4.93	137	28	0.86	121	42	1.71	91	47	2.72	208	10	0.52	156	11	1.38	194	5	0.61
50	Tiredness, general weakness	R53	A04	B		69	22	2.93	50	118	4.81	63	85	2.61	42	114	4.65	52	77	4.46	31	150	7.86	110	16	2.01	114	15	1.84
51	Gallstones	K80.-	D98		D	154	6	0.80	51	116	4.73	84	60	1.84	100	53	2.16	125	26	1.51	185	15	0.79	98	20	2.52	71	27	3.31
52	Foot deformities	M21.4/6,M20.4	L98		D	41	43	5.73	52	114	4.65	39	168	5.15	39	128	5.22	30	112	6.49	96	42	2.20	65	28	3.52	67	28	3.44
53	Paraesthesia + Polyneuropathia	R20.-	N06	A		53	33	4.40	53	108	4.40	90	56	1.72	68	86	3.51	149	18	1.04	75	57	2.99	102	19	2.39	86	22	2.70
54	Menstrual disorders	N92,94	X5,6,7,8,89,90	B		123	10	1.33	54	108	4.40	89	57	1.75	37	133	5.42	56	75	4.35	56	77	4.04	25	67	8.43	81	23	2.82
55	Prostatic hypertrophy	N40	Y85		C/D	48	37	4.93	55	106	4.32	29	278	8.53	204	11	0.45	106	35	2.03	91	45	2.36	76	25	3.15	162	8	0.98
56	Fears / Gen. Anxiety Disorder + Panic Disorder / Attack	F41.0,F41.1	P01,P74	A/B or	C	51	35	4.67	56	97	3.95	74	69	2.12	207	10	0.41	186	12	0.70	65	66	3.46	160	10	1.26	214	4	0.49
57	Nervousness	R45	P01	A		227	2	0.27	57	97	3.95	277	2	0.06	51	105	4.28	86	49	2.84	24	188	9.85	20	76	9.56	124	13	1.60
58	Insect bites	T14.0, T63	S12		C/D	79	17	2.27	58	96	3.91	45	138	4.23	35	137	5.58	34	103	5.97	48	89	4.66	40	44	5.54	57	32	3.93
59	Peripheral Arterial Vascular Disease	I70.2	K92		C/D	65	25	3.33	59	95	3.87	49	117	3.59	107	51	2.08	62	71	4.11	131	29	1.52	122	15	1.89	229	2	0.25
60	Warts + Molluca contagiosa	B07	S03,S95		C/D	85	16	2.13	60	95	3.87	78	66	2.02	44	110	4.48	17	185	10.72	21	198	10.38	41	43	5.41	73	26	3.19
61	Haematoma (incl. subconj./.subungual)	T14.0	S16		C	99	14	1.87	61	95	3.87	127	31	0.95	59	97	3.95	122	27	1.56	45	95	4.98	105	18	2.26	72	27	3.31
62	Dermatitis, acute + Photodermatoses	L30.9,L55,L59	S88,S80	A or	C	63	27	3.60	62	91	3.71	208	10	0.31	26	170	6.93	32	107	6.20	58	72	3.77	60	29	3.65	50	34	4.17
63	Tinnitus	H93.1	H03	A		131	9	1.20	63	91	3.71	99	50	1.53	82	70	2.85	134	24	1.39	133	29	1.52	88	22	2.77	163	8	0.98
64	Cervico-brachial syndrome	G56.9	L09,N05		C	17	111	14.80	64	90	3.67	60	90	2.76	73	83	3.38	33	107	6.20	118	33	1.73	42	41	5.16	39	43	5.28
65	Tick bites	T14.03	S17		D	150	7	0.93	65	87	3.55	106	47	1.44	41	116	4.73	78	57	3.30	158	21	1.10	109	17	2.14	228	2	0.25
66	Collapse / circulatory weakness +Syncope (fainting)	R55	A06	A		77	18	2.40	66	84	3.42	100	48	1.47	46	108	4.40	41	91	5.27	66	65	3.41	79	24	3.02	139	11	1.35
67	Benign skin lesions	D23	S79		C/D	119	11	1.47	67	83	3.38	177	17	0.52	70	84	3.42	48	83	4.81	33	141	7.39	49	36	4.53	84	22	2.70
68	Glaucoma	H40.0/H40.9	F93		D	144	7	0.93	68	83	3.38	51	113	3.47	158	25	1.02	216	7	0.41	269	3	0.16	304	0	0.00	0	0	0.00
69	Acne vulgaris+Acne rosacea	L70,L71	S96,S99		C/D	89	15	2.00	69	82	3.34	62	87	2.67	117	44	1.79	85	50	2.90	82	51	2.67	47	37	4.66	131	12	1.47
70	Conjunktivitis	H10.9	F70,71		C/D	45	38	5.07	70	81	3.30	26	282	8.65	24	206	8.40	46	85	4.93	49	87	4.56	18	82	10.32	15	104	12.77
71	Injury, infected	T79.3	S11		C/D	261	0	0.00	71	80	3.26	67	75	2.30	79	72	2.93	105	35	2.03	69	63	3.30	69	26	3.27	34	50	6.14
72	Laryngitis + Croup	J04.0,R49.0	R23,R77	A or	C	22	79	10.53	72	79	3.22	35	196	6.01	64	90	3.67	76	58	3.36	137	28	1.47	67	27	3.40	79	24	2.95
73	Otitis externa	H60	H70		C	46	38	5.07	73	79	3.22	54	102	3.13	55	100	4.08	53	77	4.46	51	86	4.51	100	19	2.39	80	24	2.95
74	Bronchial asthma	J45.0/1/8/9, J46	R96		C/D	33	54	7.20	74	76	3.10	36	184	5.64	90	64	2.61	93	44	2.55	62	71	3.72	31	57	7.17	41	40	4.91
75	Nausea	R11	D09	A		122	10	1.33	75	75	3.06	70	73	2.24	96	56	2.28	233	3	0.17	88	48	2.52	253	1	0.13	218	4	0.49
76	Otitis media	H66.9	H71		C	14	127	16.93	76	73	2.97	25	310	9.51	16	280	11.41	16	191	11.07	16	243	12.73	17	82	10.32	12	115	14.12
77	Thrombophlebitis superficial + Deep venous thrombosis	I80.-	K94		C/D	62	28	3.73	77	72	2.93	98	50	1.53	48	107	4.36	51	79	4.58	36	119	6.24	30	57	7.17	40	40	4.91
78	Sight / vision probles	H53.8,H53.9	F05	A		76	18	2.40	78	72	2.93	83	62	1.90	124	41	1.67	82	52	3.01	143	26	1.36	193	6	0.75	233	2	0.25
79	Swollen legs	R60	K07	A		84	16	2.13	79	72	2.93	68	75	2.30	91	63	2.57	54	77	4.46	76	55	2.88	57	30	3.77	106	16	1.96
80	Odontogenic inflammation	K04.-	D82		C/D	129	9	1.20	80	72	2.93	65	81	2.48	77	74	3.02	181	12	0.70	123	32	1.68	280	0	0.00	0	0	0.00
81	Dyspnoea	R06.0	R02	A		263	0	0.00	81	71	2.89	93	52	1.59	101	53	2.16	68	64	3.71	86	49	2.57	171	8	1.01	60	32	3.93
82	Sinus infection (all locations)	J01,J32	R75		C	9	145	19.33	82	70	2.85	33	239	7.33	36	135	5.50	31	108	6.26	30	154	8.07	44	39	4.91	44	37	4.54
83	Ulcus curis	I83.9,L97	S97		C/D	102	13	1.73	83	70	2.85	112	43	1.32	43	114	4.65	80	52	3.01	80	53	2.78	63	29	3.65	58	32	3.93
84	Abdominal cramps	R10.-	D1	A		158	6	0.80	84	66	2.69	164	20	0.61	56	99	4.04	115	31	1.80	35	121	6.34	50	36	4.53	38	47	5.77
85	Hematuria (incl. Microhematuria)	N02,R31	U06	A		176	4	0.53	85	65	2.65	140	27	0.83	173	20	0.82	50	82	4.75	106	38	1.99	172	8	1.01	248	1	0.12
86	Tender eyes and watering of the eye	H57.1,H04.2	F01,F03	A/B		295	0	0.00	86	65	2.65	265	3	0.09	155	26	1.06	159	17	0.99	240	5	0.26	125	15	1.89	230	2	0.25
87	Nikotine abuse	F17.2	P17		D	21	80	10.66	87	64	2.61	72	70	2.15	308	2	0.08	250	3	0.17	141	27	1.41	282	0	0.00	0	0	0.00

				Type of classification		Kleinbichler 2012 *n* = 7502			Fink2 2005-09 *n* = 24.541			Kasper 2005-09 *n* = 32.605			Fink1 1989-99 *n* = 24.532			Danninger 1991-96 *n* = 17.255			Landolt-Theus 1983-88 *n* = 19.082			Braun2 1977-80 n = 7.948			Braun1 1954-59 *n* = 8.146		
Rank Fink2	Results of consultation	ICD 10 codes	ICPC 2 codes	A=symptom, B=group of symptoms	C=image of a disease or D=diagnosis	Rank	n	Rate per 1000 cases (‰)	Rank	n	Rate per 1000 cases (‰)	Rank	n	Rate per 1000 cases (‰)	Rank	n	Rate per 1000 cases (‰)	Rank	n	Rate per 1000 cases (‰)	Rank	n	Rate per 1000 cases (‰)	Rank	n	Rate per 1000 cases (‰)	Rank	n	Rate per 1000 cases (‰)
88	Dysuria	R30.-	U01	A		111	12	1.60	88	64	2.61	184	16	0.49	66	87	3.55	70	63	3.65	46	94	4.93	192	6	0.75	157	9	1.10
89	Psoriasis vulgaris	L40.-	S91		D	136	8	1.07	89	64	2.61	52	109	3.34	97	56	2.28	151	18	1.04	177	16	0.84	97	20	2.52	220	3	0.37
90	Blood on/ in faeces	K62.5	D16	A		88	16	2.13	90	63	2.57	133	29	0.89	80	71	2.89	128	25	1.45	174	17	0.89	153	11	1.38	231	2	0.25
91	Sprain/strain of joint NOS	S03,S43,S53,S63,S73	L79		C	109	12	1.60	91	63	2.57	94	52	1.59	84	69	2.81	67	65	3.77	32	145	7.60	59	29	3.65	107	16	1.96
92	Naevus/mole	D22	S82		C/D	39	44	5.87	92	61	2.49	152	23	0.71	112	46	1.88	141	20	1.16	100	40	2.10	91	21	2.64	207	4	0.49
93	Dermatophytosis	B35,B36	S74		C/D	74	19	2.53	93	60	2.44	64	81	2.48	38	132	5.38	47	83	4.81	42	101	5.29	26	66	8.30	29	57	7.00
94	Laceration (Excoriation	T14.0	S17		D	170	4	0.53	94	60	2.44	116	41	1.26	85	69	2.81	99	39	2.26	93	44	2.31	138	13	1.64	53	34	4.17
95	Pregnancy	Z34	W82		D	75	18	2.40	95	59	2.40	55	100	3.07	52	103	4.20	36	99	5.74	114	35	1.83	52	36	4.53	112	15	1.84
96	Pruritus, general	L29.8,L29.9	S02	A		214	2	0.27	96	58	2.36	132	30	0.92	109	48	1.96	188	11	0.64	111	36	1.89	142	12	1.51	166	8	0.98
97	Alcohol abuse	F10	P15,P16		D	96	15	2.00	97	56	2.28	183	16	0.49	103	52	2.12	120	28	1.62	110	37	1.94	55	31	3.90	126	13	1.60
98	Benign mammary dysplasia	N63	X19		C/D	132	9	1.20	98	56	2.28	143	25	0.77	63	93	3.79	63	70	4.06	182	15	0.79	143	12	1.51	155	9	1.10
99	Urinary calculus	N20,N21	U95		D	157	6	0.80	99	56	2.28	156	22	0.67	92	63	2.57	95	42	2.43	67	65	3.41	61	29	3.65	153	9	1.10
100	Memory disturbance	R41	P20	A		0	0	0.00	100	56	2.28	0	0	0.00	216	9	0.37	178	14	0.81	216	9	0.47	0	0	0.00	0	0	0.00
101	Thyreoiditis Hashimoto /Thyreoiditis de-Quervain	E06.1,E06.3	T99		D	55	32	4.27	101	54	2.20	97	52	1.59	347	1	0.04	224	7	0.41	282	2	0.10	270	1	0.13	0	0	0.00
102	Urticaria	L50.-	S98		C	134	9	1.20	102	54	2.20	147	23	0.71	60	97	3.95	133	24	1.39	148	24	1.26	77	25	3.15	68	28	3.44
103	Meteorism	R14	D08	A		172	4	0.53	103	54	2.20	121	37	1.13	139	33	1.35	132	25	1.45	92	45	2.36	86	22	2.77	171	7	0.86
104	Ectopic beats	I49.4	K05,K80	A		215	2	0.27	104	53	2.16	159	22	0.67	130	36	1.47	210	8	0.46	125	32	1.68	173	8	1.01	0	0	0.00
105	Onychomycosis	B35.1	S74		C/D	155	6	0.80	105	52	2.12	146	24	0.74	125	41	1.67	118	29	1.68	214	9	0.47	188	7	0.88	0	0	0.00
106	Parkinsonism+Tremor,unspecific	G20,21,G25.0,R25.1	N87,N08	A or	C	108	12	1.60	106	51	2.08	80	64	1.96	93	62	2.53	145	19	1.10	154	22	1.15	115	16	2.01	172	7	0.86
107	Vitium cordis	I38	K71		D	60	28	3.73	107	50	2.04	53	104	3.19	191	14	0.57	218	7	0.41	194	14	0.73	259	1	0.13	182	7	0.86
108	Ingrowing toe nail+Paronychia	L60.0,L03.0	S94,S09		C/D	90	15	2.00	108	50	2.04	71	72	2.21	49	106	4.32	66	67	3.88	44	95	4.98	94	20	2.52	19	83	10.19
109	Chickenpox	B01	A72		C	138	8	1.07	109	50	2.04	88	57	1.75	65	88	3.59	89	48	2.78	98	41	2.15	139	13	1.64	56	33	4.05
110	Kidney dysfunction/ Nephropathy	N17,18,19	U99		D	38	44	5.87	110	49	2.00	59	93	2.85	227	7	0.29	138	22	1.27	167	19	1.00	194	6	0.75	234	2	0.25
111	Chronic polyarthritis (Rheumatoid arthritis)	M05,06	L88		D	61	28	3.73	111	49	2.00	91	55	1.69	159	25	1.02	238	3	0.17	144	26	1.36	83	24	3.02	51	34	4.17
112	Vaginitis+Genital candidiasis female	N76,B37.3	X84,X72		C/D	116	11	1.47	112	49	2.00	47	121	3.71	61	97	3.95	29	121	7.01	113	35	1.83	95	20	2.52	165	8	0.98
113	Haemorrhoids	K64.-	K96		D	130	9	1.20	113	49	2.00	81	63	1.93	88	65	2.65	94	42	2.43	60	72	3.77	46	37	4.66	117	14	1.72
114	Heel complaint (Calcaneodynia)	M77.9,M79.07	L17	A		103	13	1.73	114	48	1.96	122	36	1.10	134	34	1.39	136	22	1.27	134	29	1.52	212	4	0.50	198	5	0.61
115	Erythema migrans (tickborne)	A69.2	A78		C/D	121	10	1.33	115	48	1.96	56	98	3.01	104	52	2.12	114	32	1.85	0	0	0.00	0	0	0.00	0	0	0.00
116	Adverse effect or poisoning by medical agent	T50,Y57	A84,A85		C/D	92	15	2.00	116	47	1.92	82	62	1.90	102	53	2.16	81	52	3.01	52	84	4.40	118	15	1.89	209	4	0.49
117	Carpal tunnel syndrome	G56.0	N93		D	137	8	1.07	117	46	1.87	86	59	1.81	156	26	1.06	137	22	1.27	215	9	0.47	261	1	0.13	204	5	0.61
118	Fits, non-specific	R69	A29	A/B		0	0	0.00	118	46	1.87	149	23	0.71	179	19	0.77	255	2	0.12	135	29	1.52	150	11	1.38	102	18	2.21
119	Herpes simplex (cold sore)	B00.1	S71		C	87	16	2.13	119	45	1.83	117	41	1.26	71	84	3.42	60	73	4.23	153	22	1.15	84	23	2.89	98	19	2.33
120	Aphtous ulcers	K12.0,B00.2	D83		C	139	8	1.07	120	45	1.83	124	32	0.98	69	86	3.51	97	40	2.32	103	39	2.04	71	26	3.27	43	38	4.66
121	Sexual problems	F52,N48.4	P07,08,Y07	B		114	12	1.60	121	44	1.79	251	5	0.15	167	22	0.90	183	12	0.70	151	23	1.21	186	7	0.88	232	2	0.25
122	Distorsio pedis	S93.4	L77		C	146	7	0.93	122	44	1.79	77	66	2.02	76	76	3.10	64	68	3.94	23	192	10.06	38	45	5.66	48	35	4.30
123	Stroke	G45.9,G46,I63,I64	K90		D	30	59	7.86	123	43	1.75	165	20	0.61	67	87	3.55	26	142	8.23	72	59	3.09	80	24	3.02	54	33	4.05
124	Tennis elbow	M77.1	L93		C	64	26	3.47	124	43	1.75	73	69	2.12	94	62	2.53	110	33	1.91	61	72	3.77	201	5	0.63	111	16	1.96
125	Nervous breakdown, burnout	F43	P02		C	97	14	1.87	125	43	1.75	105	47	1.44	143	31	1.26	236	3	0.17	152	23	1.21	151	11	1.38	193	5	0.61
126	Epistaxis	R04.0	R06	A or	D	166	5	0.67	126	43	1.75	120	37	1.13	108	51	2.08	130	25	1.45	95	43	2.25	111	16	2.01	61	31	3.81
127	Eczema, seborrhoeic	L21.-	S86		C	211	2	0.27	127	43	1.75	96	52	1.59	128	39	1.59	71	63	3.65	169	18	0.94	103	19	2.39	140	11	1.35
128	Migraine	G43.-	N89		C	57	30	4.00	128	42	1.71	46	129	3.96	129	37	1.51	182	12	0.70	115	35	1.83	140	13	1.64	192	5	0.61
129	Lymphadenitis	L04,I88	B70,71	A or	C	66	23	3.07	129	42	1.71	110	45	1.38	135	34	1.39	168	15	0.87	309	1	0.05	182	8	1.01	146	11	1.35
130	Pollakisuria	R45	U02	A		195	3	0.40	130	41	1.67	210	10	0.31	153	27	1.10	142	20	1.16	191	14	0.73	72	26	3.27	100	18	2.21
131	Loss of weight	R63.4	T08	A		270	0	0.00	131	41	1.67	161	21	0.64	126	40	1.63	152	18	1.04	164	20	1.05	256	1	0.13	130	13	1.60
132	Swelling and infiltration, unspecific	R22	A08, S04	A		113	12	1.60	132	40	1.63	224	8	0.25	144	31	1.26	59	75	4.35	43	95	4.98	64	28	3.52	183	6	0.74
133	Bursitis acuta	M70,71	L87		C/D	145	7	0.93	133	39	1.59	66	78	2.39	131	36	1.47	75	60	3.48	136	28	1.47	96	20	2.52	113	15	1.84
134	Herpes zoster/ Shingles	B02, B02.9	S70		C/D	149	7	0.93	134	39	1.59	103	47	1.44	98	56	2.28	129	25	1.45	104	39	2.04	183	7	0.88	96	20	2.46
135	Otalgia	H92.0	H01	A		100	14	1.87	135	38	1.55	148	23	0.71	53	103	4.20	124	26	1.51	116	34	1.78	108	17	2.14	87	22	2.70
136	Eustachian salpingitis	H68	H73		C	135	9	1.20	136	36	1.47	170	19	0.58	113	46	1.88	162	16	0.93	81	53	2.78	170	8	1.01	47	37	4.54
137	Tachycardia	R00.0	K04	A		159	6	0.80	137	36	1.47	178	17	0.52	217	9	0.37	173	15	0.87	160	21	1.10	123	15	1.89	94	20	2.46
138	Pruritus, localized	L29.8	S02	A		185	4	0.53	138	36	1.47	235	7	0.21	266	4	0.16	113	33	1.91	176	16	0.84	154	11	1.38	257	0	0.00
139	Epilepsy	G40,G41	N88		C	86	16	2.13	139	35	1.43	92	54	1.66	149	28	1.14	169	15	0.87	156	22	1.15	131	14	1.76	104	17	2.09
140	Joint replacement, St.p. with complaints	T84.-	A89		C/D	117	11	1.47	140	35	1.43	50	115	3.53	169	21	0.86	202	9	0.52	232	6	0.31	228	3	0.38	0	0	0.00
141	Lumbar spinal stenosis	M48.-	L83,84		D	148	7	0.93	141	35	1.43	101	48	1.47	0	0	0.00	0	0	0.00	0	0	0.00	0	0	0.00	0	0	0.00
142	Eczema, anogenital	L30.4	S87		C	183	4	0.53	142	35	1.43	225	8	0.25	136	34	1.39	58	75	4.35	97	41	2.15	112	16	2.01	191	5	0.61
143	Eczema, dyshidrotic	L30.1	S92		C	193	3	0.40	143	35	1.43	191	14	0.43	150	28	1.14	146	19	1.10	253	4	0.21	169	9	1.13	128	13	1.60
144	Sore throat	R07.0	R74	A		44	39	5.20	144	34	1.39	0	0	0.00	45	110	4.48	37	98	5.68	37	112	5.87	53	33	4.15	69	27	3.31
145	Psychosis, chronic	F20.5	P71,P72,P73		C	110	12	1.60	145	34	1.39	162	21	0.64	170	21	0.86	184	12	0.70	117	34	1.78	66	27	3.40	170	7	0.86
146	Callosity	L84	20		D	125	10	1.33	146	34	1.39	128	31	0.95	72	84	3.42	101	38	2.20	224	7	0.37	134	14	1.76	95	20	2.46
147	Sweating, general	R61.1	A09	A		247	1	0.13	147	33	1.34	223	8	0.25	168	22	0.90	176	14	0.81	193	14	0.73	215	4	0.50	158	9	1.10
148	Restless legs	G25.81	L19	B		124	10	1.33	148	32	1.30	115	42	1.29	348	1	0.04	0	0	0.00	265	4	0.21	0	0	0.00	0	0	0.00
149	Inguinal or femural hernia	K40.-,K41,-	D89,D91		D	151	7	0.93	149	32	1.30	131	30	0.92	127	40	1.63	65	68	3.94	87	48	2.52	70	26	3.27	42	40	4.91
150	Alopecia diffusa	L65.-	S23	A		167	5	0.67	150	32	1.30	169	20	0.61	137	34	1.39	116	30	1.74	198	13	0.68	117	16	2.01	208	4	0.49
151	Skin cancer	C44.-	S77		D	82	17	2.27	151	30	1.22	205	11	0.34	142	32	1.30	153	18	1.04	200	12	0.63	177	8	1.01	175	7	0.86
152	Bites	T14.1	S13		D	162	6	0.80	152	29	1.18	256	4	0.12	154	27	1.10	175	14	0.81	89	47	2.46	128	14	1.76	62	31	3.81
153	Ruptured tendon	S46-S96,T14.-,M66.-	L81		D	217	2	0.27	153	29	1.18	173	18	0.55	234	6	0.24	213	8	0.46	162	21	1.10	255	1	0.13	160	9	1.10
154	Pyogenic infection, other	L08.0	S76		C/D	0	0	0.00	154	28	1.14	69	74	2.27	120	43	1.75	196	10	0.58	124	32	1.68	148	11	1.38	213	4	0.49
155	Hyperkeratosis	L85.9	S80		D	0	0	0.00	155	28	1.14	211	10	0.31	174	20	0.82	165	16	0.93	145	25	1.31	238	2	0.25	181	7	0.86
156	Skin disease, other	L98.9	S99		C	70	22	2.93	156	27	1.10	192	14	0.43	75	79	3.22	214	7	0.41	139	28	1.47	175	8	1.01	145	11	1.35
157	Meniscus lesion	S83.2,M23.2-3	L96,L99		C/D	118	11	1.47	157	27	1.10	130	31	0.95	221	8	0.33	193	11	0.64	129	31	1.62	225	3	0.38	240	2	0.25
158	Cholecystopathy (incl. Cholangiopathy)	K82.9,K83.9	D98	B or	C	173	4	0.53	158	27	1.10	126	31	0.95	171	21	0.86	170	15	0.87	102	40	2.10	129	14	1.76	46	37	4.54
159	Ulcerative colitis/Chronic enteritis	K50,K51	D94		D	71	21	2.80	159	26	1.06	181	17	0.52	200	12	0.49	239	3	0.17	257	4	0.21	235	3	0.38	242	2	0.25
160	Muscle distorsion, muscle tear	S09.1,S16,S46-S96, T14.6,M61.1,M62.6	L19,L81		C/D	120	11	1.47	160	26	1.06	199	12	0.37	184	17	0.69	172	15	0.87	71	62	3.25	74	25	3.15	59	32	3.93
161	Lipoma	D17.-	S78		C/D	216	2	0.27	161	26	1.06	166	20	0.61	189	15	0.61	217	7	0.41	163	21	1.10	152	11	1.38	133	12	1.47
162	Glossodynia / tongue burning (incl. Glossitis)	K14.6	D20	A		271	0	0.00	162	26	1.06	168	20	0.61	222	8	0.33	244	3	0.17	203	12	0.63	240	2	0.25	189	6	0.74
163	Injury, combined not severe	T00.9	S19		C/D	28	67	8.93	163	25	1.02	87	58	1.78	163	24	0.98	35	103	5.97	34	137	7.18	37	47	5.91	24	66	8.10
164	Erysipelas	A46	S76		C	80	17	2.27	164	25	1.02	139	27	0.83	115	45	1.83	102	38	2.20	231	6	0.31	199	6	0.75	168	8	0.98
165	Sebaceous cyst	L72.1	S93		D	94	15	2.00	165	25	1.02	119	39	1.20	132	35	1.43	77	58	3.36	142	26	1.36	130	14	1.76	141	11	1.35
166	Rash	R21	S07	A		127	10	1.33	166	25	1.02	158	22	0.67	105	52	2.12	49	82	4.75	119	33	1.73	120	15	1.89	210	4	0.49
167	Hernia umbilicalis	K42.9	D91		D	180	4	0.53	167	25	1.02	216	9	0.28	185	17	0.69	203	9	0.52	233	6	0.31	179	8	1.01	110	16	1.96
168	Problems in the family	Z60.1/9,Z62.9,Z63.1	Z20,Z21	A		209	3	0.40	168	24	0.98	0	0	0.00	0	0	0.00	422	0	0.00	126	32	1.68	0	0	0.00	0	0	0.00
169	Raynaud syndrome	I73.0	K92		C	244	1	0.13	169	24	0.98	213	10	0.31	267	4	0.16	0	0	0.00	248	5	0.26	206	5	0.63	0	0	0.00
170	Polycythaemia rubra vera	D75.0	B99		D	301	0	0.00	170	24	0.98	273	3	0.09	192	14	0.57	0	0	0.00	344	0	0.00	0	0	0.00	0	0	0.00
171	Scarlet fever	A38	A78		C/D	81	17	2.27	171	23	0.94	197	13	0.40	122	42	1.71	131	25	1.45	186	15	0.79	0	0	0.00	0	0	0.00
172	Pancreatic disorder	K86.9	D99		C	184	4	0.53	172	23	0.94	233	7	0.21	235	6	0.24	263	2	0.12	237	6	0.31	205	5	0.63	223	3	0.37
173	Diapers rash	L22	S89		D	190	3	0.40	173	23	0.94	157	22	0.67	118	44	1.79	215	7	0.41	255	4	0.21	208	5	0.63	196	5	0.61
174	Combustion / scalding	T20-T28,T29,T30	S14		D	235	1	0.13	174	23	0.94	108	46	1.41	138	34	1.39	117	30	1.74	83	51	2.67	90	21	2.64	76	25	3.07
175	Ectopic beats /palpitations	R00.2	K04	A		78	18	2.40	175	22	0.90	190	14	0.43	110	48	1.96	234	3	0.17	146	25	1.31	167	9	1.13	120	14	1.72
176	Skoliosis	M41.-	L85		D	101	14	1.87	176	22	0.90	175	18	0.55	349	1	0.04	135	23	1.33	324	0	0.00	0	0	0.00	0	0	0.00
177	Hypertrophy tonsils/adenoids	J35.3,J35.8	R90		C	169	5	0.67	177	22	0.90	250	5	0.15	201	12	0.49	156	18	1.04	178	16	0.84	195	6	0.75	186	6	0.74
178	Eczema palmar/plantar	L30.9,L23,24,25	S88		C/D	197	3	0.40	178	22	0.90	215	9	0.28	157	26	1.06	84	51	2.96	57	74	3.88	68	26	3.27	45	37	4.54
179	Pruritus analis / anogenitalis	L29.3	D05	A		199	3	0.40	179	21	0.86	230	7	0.21	133	35	1.43	235	3	0.17	140	27	1.41	114	16	2.01	148	10	1.23
180	Incisional hernia	K43.-	D91		C/D	213	2	0.27	180	21	0.86	125	32	0.98	228	7	0.29	205	9	0.52	187	15	0.79	258	1	0.13	251	1	0.12
181	Problems working place	Z56.-	Z05	A		230	2	0.27	181	21	0.86	0	0	0.00	287	3	0.12	0	0	0.00	157	22	1.15	0	0	0.00	0	0	0.00
182	Skin scar, painful	L90.5	S82	A		0	0	0.00	182	21	0.86	290	1	0.03	223	8	0.33	103	38	2.20	184	15	0.79	144	12	1.51	184	6	0.74

Rank Fink2	Results of consultation	ICD 10 codes	ICPC 2 codes	Type of classification: A=symptom, B=group of symptoms	C=image of a disease or D=diagnosis	Kleinbichler 2012 *n* = 7502			Fink2 2005-09 *n* = 24.541			Kasper 2005-09 *n* = 32.605			Fink1 1989-99 *n* = 24.532			Danninger 1991-96 *n* = 17.255			Landolt-Theus 1983-88 *n* = 19.082			Braun2 1977-80 n = 7.948			Braun1 1954-59 *n* = 8.146		
						Rank	n	Rate per 1000 cases (‰)	Rank	n	Rate per 1000 cases (‰)	Rank	n	Rate per 1000 cases (‰)	Rank	n	Rate per 1000 cases (‰)	Rank	n	Rate per 1000 cases (‰)	Rank	n	Rate per 1000 cases (‰)	Rank	n	Rate per 1000 cases (‰)	Rank	n	Rate per 1000 cases (‰)
183	Abscess / carbuncle	L02.-	S10		C/D	67	23	3.07	183	20	0.81	113	42	1.29	119	44	1.79	121	28	1.62	77	55	2.88	36	49	6.17	7	135	16.57
184	Lymphnode(s) enlarged, non-specific	R59.-	B02	A		153	7	0.93	184	20	0.81	203	11	0.34	99	56	2.28	104	36	2.09	84	50	2.62	136	13	1.64	91	21	2.58
185	Wound puncture (Vln.ictus)	S01-S91,T14.1	S19		D	264	0	0.00	185	20	0.81	102	47	1.44	145	30	1.22	174	14	0.81	105	39	2.04	87	22	2.77	93	20	2.46
186	Problems with partner	Z63.0	Z12	A		359	0	0.00	186	20	0.81	348	0	0.00	193	14	0.57	0	0	0.00	90	46	2.41	0	0	0.00	0	0	0.00
187	Chronic pain syndrome	R52.2	A1	B		434	0	0.00	187	20	0.81	0	0	0.00	0	0	0.00	0	0	0.00	0	0	0.00	0	0	0.00	0	0	0.00
188	Diverticulitis	K57.-	D92		C/D	112	12	1.60	188	18	0.73	186	16	0.49	309	2	0.08	251	3	0.17	245	5	0.26	0	0	0.00	0	0	0.00
189	Distorsio genus	S83.6	L78		C	171	4	0.53	189	18	0.73	118	41	1.26	140	33	1.35	126	26	1.51	68	65	3.41	126	14	1.76	127	13	1.60
190	Behavioral disorders	F90-F94	P22		C	218	2	0.27	190	18	0.73	176	18	0.55	236	6	0.24	264	2	0.12	0	0	0.00	0	0	0.00	0	0	0.00
191	Globus, lump in the throat	F45.8	R21,P75	A		219	2	0.27	191	18	0.73	185	16	0.49	151	28	1.14	211	8	0.46	188	15	0.79	204	5	0.63	177	7	0.86
192	Cold feet	R68.8	A02	A		229	2	0.27	192	18	0.73	0	0	0.00	218	9	0.37	204	9	0.52	202	12	0.63	133	14	1.76	211	4	0.49
193	Descensus (Prolaps) vaginae (et uteri)	N81.-	X87		D	279	0	0.00	193	18	0.73	202	11	0.34	224	8	0.33	143	20	1.16	207	11	0.58	141	13	1.64	75	26	3.19
194	Injuries, others	S00.-to T14.-	S19		C/D	35	48	6.40	194	17	0.69	136	28	0.86	166	23	0.94	160	17	0.99	109	38	1.99	92	21	2.64	164	8	0.98
195	Impetigo contagiosa	L01.0	S84		C	98	14	1.87	195	17	0.69	111	44	1.35	147	29	1.18	119	29	1.68	122	32	1.68	34	53	6.67	22	67	8.22
196	Stomatitis non-specific.+Candida-Stomatitis (oral thru	K12.1,B37.0	D83	A or	C	147	7	0.93	196	17	0.69	79	64	1.96	116	45	1.83	98	40	2.32	223	7	0.37	189	7	0.88	151	10	1.23
197	Sleep apnea syndrome	G47.3	P03		C/D	152	7	0.93	197	17	0.69	141	27	0.83	288	3	0.12	275	2	0.12	0	0	0.00	0	0	0.00	0	0	0.00
198	Trigger finger	M65.3	L87		D	274	0	0.00	198	17	0.69	189	15	0.46	208	10	0.41	241	3	0.17	258	4	0.21	220	4	0.50	237	2	0.25
199	Angular cheilitis (Perlèche)	K13.0	S76		C/D	178	4	0.53	199	16	0.65	179	17	0.52	209	10	0.41	191	11	0.64	310	1	0.05	190	7	0.88	147	11	1.35
200	Nail deformities and dystrophies	L60.2-60.8,9	S99	A or	D	208	3	0.40	200	16	0.65	0	0	0.00	237	6	0.24	221	7	0.41	227	7	0.37	264	1	0.13	252	1	0.12
201	Ecchymosis	R23.3	A10,S29	A		246	1	0.13	201	16	0.65	222	8	0.25	152	28	1.14	237	3	0.17	312	1	0.05	200	6	0.75	137	12	1.47
202	Dysphagia	R13	D21	A		0	0	0.00	202	16	0.65	145	25	0.77	350	1	0.04	252	3	0.17	211	10	0.52	289	0	0.00	247	2	0.25
203	Postcholecystectomy syndrome	K91.5	D99	B		0	0	0.00	203	16	0.65	0	0	0.00	268	4	0.16	227	5	0.29	235	6	0.31	107	18	2.26	132	12	1.47
204	Tendinitis	M65.9	L87		C	107	12	1.60	204	15	0.61	48	118	3.62	87	66	2.69	140	21	1.22	50	87	4.56	159	10	1.26	134	12	1.47
205	Foreign body in skin/under nail	T14.0,T14.1	S15		D	177	4	0.53	205	15	0.61	171	19	0.58	123	42	1.71	112	33	1.91	128	31	1.62	174	8	1.01	109	16	1.96
206	Attention deficit syndrome	F90.0	P81	B		202	3	0.40	206	15	0.61	244	6	0.18	0	0	0.00	0	0	0.00	0	0	0.00	0	0	0.00	0	0	0.00
207	Gingivitis	K05.0	D82		C	245	1	0.13	207	15	0.61	217	9	0.28	180	19	0.77	177	14	0.81	171	18	0.94	161	10	1.26	143	11	1.35
208	Decubitus ulcer (bedsore)	L89.-	S97		D	250	1	0.13	208	15	0.61	253	5	0.15	160	25	1.02	0	0	0.00	0	0	0.00	349	0	0.00	0	0	0.00
209	Leukopenia non-specific	D70.7	B84	A		0	0	0.00	209	15	0.61	274	3	0.09	310	2	0.08	0	0	0.00	0	0	0.00	0	0	0.00	0	0	0.00
210	Appendicitis	K35, K37	D88		C/D	104	13	1.73	210	14	0.57	207	10	0.31	148	29	1.18	164	16	0.93	94	44	2.31	81	24	3.02	30	57	7.00
211	Infertility/subfertility problems	Z31.-	W15	B		201	3	0.40	211	14	0.57	241	6	0.18	202	12	0.49	259	2	0.12	204	12	0.63	241	2	0.25	0	0	0.00
212	Conjunktivitis after foreign body or after phys./chem.contact	H10.8, T26.1,T26.6	F76, F79		C/D	237	1	0.13	212	14	0.57	142	26	0.80	141	33	1.35	179	13	0.75	149	24	1.26	89	22	2.77	70	27	3.31
213	Refractive error	H52.-	F91		D	272	0	0.00	213	14	0.57	182	17	0.52	351	1	0.04	69	64	3.71	252	4	0.21	207	5	0.63	0	0	0.00
214	Hallux malleus (mallet toe)	M20.4	L98		D	281	0	0.00	214	14	0.57	227	8	0.25	246	5	0.20	245	3	0.17	228	7	0.37	265	1	0.13	0	0	0.00
215	Phimosis	N47	Y81		D	128	10	1.33	215	13	0.53	206	11	0.34	210	10	0.41	242	3	0.17	196	14	0.73	187	7	0.88	215	4	0.49
216	Blepharitis Blepharoconjunktivitis	H01.0,H10.5	F72		C	192	3	0.40	216	13	0.53	188	15	0.46	164	24	0.98	96	41	2.38	279	2	0.10	247	2	0.25	225	3	0.37
217	Hallux valgus	M20.1	L98		D	223	2	0.27	217	13	0.53	234	7	0.21	205	11	0.45	240	3	0.17	165	20	1.05	239	2	0.25	243	2	0.25
218	Enuresis nocturna	F98.0	P12		C	241	1	0.13	218	13	0.53	193	14	0.43	197	13	0.53	190	11	0.64	225	7	0.37	168	9	1.13	174	7	0.86
219	Foreign body in apertures	T16,17.-18.-19.-,	76,R87,D79,U80,X82		D	0	0	0.00	219	13	0.53	187	15	0.46	203	12	0.49	161	17	0.99	201	12	0.63	164	10	1.26	144	11	1.35
220	Keloid	L91.0	S99		D	0	0	0.00	220	13	0.53	333	0	0.00	247	5	0.20	222	7	0.41	234	6	0.31	229	3	0.38	241	2	0.25
221	Injuries severe, combined	S00.-to T14.-	A81		D	126	10	1.33	221	12	0.49	150	23	0.71	238	6	0.24	107	35	2.03	107	38	1.99	184	7	0.88	129	13	1.60
222	Ganglion	M67.4	L87		C/D	140	8	1.07	222	12	0.49	172	19	0.58	194	14	0.57	155	18	1.04	155	22	1.15	176	8	1.01	121	14	1.72
223	Heart failure, acute	I50.0,I50.1	K77		C	187	3	0.40	223	12	0.49	129	31	0.95	161	25	1.02	88	49	2.84	206	11	0.58	178	8	1.01	135	12	1.47
224	Posture and gait abnormality	R29.8	L27,L29	A/B		239	1	0.13	224	12	0.49	167	20	0.61	195	14	0.57	257	2	0.12	147	25	1.31	226	3	0.38	188	6	0.74
225	Borreliosis (except Erythema migrans)	A69.2	A78		D	0	0	0.00	225	12	0.49	198	13	0.40	175	20	0.82	212	8	0.46	311	1	0.05	0	0	0.00	0	0	0.00
226	Renal cyst	N28.1	U99		C/D	0	0	0.00	226	12	0.49	245	6	0.18	425	0	0.00	0	0	0.00	426	0	0.00	0	0	0.00	0	0	0.00
227	Hallux valgus (bunion)	Q78.6	L82		D	0	0	0.00	227	12	0.49	267	3	0.09	311	2	0.08	279	2	0.12	316	1	0.05	191	7	0.88	0	0	0.00
228	Loss of appetite	R63.0	T03	A		83	17	2.27	228	11	0.45	276	3	0.09	0	0	0.00	0	0	0.00	427	0	0.00	0	0	0.00	0	0	0.00
229	Myocardial infarction, Acute Coronary Syndrome	I21.9, I24.9	K75,74		C/D	105	13	1.73	229	11	0.45	239	6	0.18	162	25	1.02	108	34	1.97	73	58	3.04	119	15	1.89	154	9	1.10
230	Hordeolum (stye)	H00.0	F92		C/D	156	6	0.80	230	11	0.45	154	22	0.67	176	20	0.82	254	2	0.12	166	20	1.05	132	14	1.76	49	35	4.30
231	Exposure to sick family member	Z60	P25		D	164	6	0.80	231	11	0.45	434	0	0.00	0	0	0.00	0	0	0.00	428	0	0.00	0	0	0.00	428	0	0.00
232	Cirrhosis of the liver	K74.6,K74.-,K70.3	D97		C/D	182	4	0.53	232	11	0.45	219	9	0.28	289	3	0.12	0	0	0.00	277	3	0.16	157	11	1.38	185	6	0.74
233	Joint dislocation / luxation	S03-T14,M22.-	L80		D	0	0	0.00	233	11	0.45	200	12	0.37	211	10	0.41	192	11	0.64	112	36	1.89	237	2	0.25	169	8	0.98
234	Drug and substance abuse	F11-F19,Z72.2	P18,P19		C/D	0	0	0.00	234	11	0.45	242	6	0.18	312	2	0.08	0	0	0.00	180	16	0.84	0	0	0.00	0	0	0.00
235	Endometriosis	N80.-	X99		C/D	0	0	0.00	235	11	0.45	260	4	0.12	352	1	0.04	292	2	0.12	288	2	0.10	0	0	0.00	0	0	0.00
236	Cardiomyopathy	I42.-	K84		C/D	54	33	4.40	236	10	0.41	160	22	0.67	0	0	0.00	0	0	0.00	0	0	0.00	0	0	0.00	0	0	0.00
237	Balanitis / Balanoposthitis	N48.1	Y75		C/D	95	15	2.00	237	10	0.41	163	21	0.64	198	13	0.53	225	5	0.29	168	19	1.00	257	1	0.13	203	5	0.61
238	Polymorph heart complaints	R07.4	K24,K01,02,04	B		238	1	0.13	238	10	0.41	155	22	0.67	86	68	2.77	150	18	1.04	108	38	1.99	82	24	3.02	118	14	1.72
239	Quincke's edema	T78.3	A92		C	0	0	0.00	239	10	0.41	196	13	0.40	206	11	0.45	260	2	0.12	212	10	0.52	243	2	0.25	202	5	0.61
240	Phantom pain	G54.6	N94		C	0	0	0.00	240	10	0.41	246	6	0.18	0	0	0.00	0	0	0.00	0	0	0.00	0	0	0.00	0	0	0.00
241	Dysgeusia / parageusia	R43.2	N16	A		0	0	0.00	241	10	0.41	281	2	0.06	225	8	0.33	261	2	0.12	230	7	0.37	227	3	0.38	200	5	0.61
242	Eye pain	H57.1	F01	A		0	0	0.00	242	10	0.41	0	0	0.00	248	5	0.20	200	10	0.58	161	21	1.10	254	1	0.13	250	1	0.12
243	Liver disease NOS	K70,K76.-	D97		C	47	38	5.07	243	9	0.37	271	3	0.09	269	4	0.16	72	63	3.65	278	2	0.10	0	0	0.00	0	0	0.00
244	Threadworms	B80	D96		D	194	3	0.40	244	9	0.37	195	13	0.40	111	47	1.92	90	47	2.72	213	9	0.47	106	18	2.26	103	17	2.09
245	Pediculosis/skin infestation	B85	S73		D	203	3	0.40	245	9	0.37	247	6	0.18	0	0	0.00	0	0	0.00	0	0	0.00	0	0	0.00	0	0	0.00
246	Gynecomastia	N62	Y16	A		206	3	0.40	246	9	0.37	255	5	0.15	0	0	0.00	0	0	0.00	0	0	0.00	0	0	0.00	0	0	0.00
247	Ossalgia (Periostalgia)	M89.9	L29	A		220	2	0.27	247	9	0.37	212	10	0.31	186	17	0.69	198	10	0.58	47	91	4.77	99	19	2.39	190	5	0.61
248	Foreign body in cornea	T15.0	F76,F79		D	236	1	0.13	248	9	0.37	109	45	1.38	188	16	0.65	123	27	1.56	170	18	0.94	54	32	4.03	78	24	2.95
249	Parotitis	K11.9	D83		C	249	1	0.13	249	9	0.37	238	7	0.21	432	0	0.00	0	0	0.00	0	0	0.00	0	0	0.00	0	0	0.00
250	Chalazion	H00.1	F72		C/D	251	1	0.13	250	9	0.37	279	2	0.06	270	4	0.16	246	3	0.17	236	6	0.31	230	3	0.38	179	7	0.86
251	Cruciate ligament injury	M23.51,23.52	L99		D	0	0	0.00	251	9	0.37	262	4	0.12	353	1	0.04	0	0	0.00	0	0	0.00	0	0	0.00	0	0	0.00
252	Unspecified abortion	O06.-	W82		D	0	0	0.00	252	9	0.37	278	2	0.06	249	5	0.20	166	16	0.93	241	5	0.26	180	8	1.01	136	12	1.47
253	Snorring	R06.5	R04	A		0	0	0.00	253	9	0.37	294	1	0.03	354	1	0.04	0	0	0.00	300	2	0.10	251	2	0.25	0	0	0.00
254	Hyperbilirubinemia	R17.-	T99	A		0	0	0.00	254	9	0.37	311	1	0.03	0	0	0.00	0	0	0.00	0	0	0.00	0	0	0.00	0	0	0.00
255	Bacteriuria	N39.0	U71	A		0	0	0.00	255	9	0.37	0	0	0.00	0	0	0.00	0	0	0.00	0	0	0.00	0	0	0.00	0	0	0.00
256	Dry skin	R23.4	S21	A		0	0	0.00	256	9	0.37	0	0	0.00	0	0	0.00	0	0	0.00	0	0	0.00	0	0	0.00	0	0	0.00
				**81**	**161**		6974	929.62		23389	953.06		31587	968.78		23676	965.11		15729	911.56		18196	953.57		7294	917.72		6888	845.57

A = symptom **B** = symptom cluster

C = image/picture of a disease **D** = sound, proven diagnosis

14 both symptom classification or diagnosis possible
