# Supplementary material for: Frequency distribution of health disorders in primary care—its consistency and meaning for diagnostics and nomenclature
Source: Wien Med Wochenschr. 2024 Jul 22;175(5-6):99–109. doi: 10.1007/s10354-024-01049-5 (PMC11928369; doi:10.1007/s10354-024-01049-5)
Supplement: Supplementary file 3 — Supplementary_material_3_corOverallyearly_correlation_matrix. Spearman correlation coefficient matrix for 21 individual statistical years [file 10354_2024_1049_MOESM3_ESM.pdf]

	mFink1_1	mFink1_2	mFink1_3	mFink1_4	mFink1_5	mFink1_6	mFink1_7	mFink1_8	mFink1_9	mFink1_10	mFink2_1	mFink2_2	mFink2_3	mFink2_4	mFink2_5	mKasper1	mKasper2	mKasper3	mKasper4	mKasper5	mKleinb
mFink1_1	1																				
mFink1_2	0.85	1																			
mFink1_3	0.83	0.84	1																		
mFink1_4	0.81	0.82	0.82	1																	
mFink1_5	0.77	0.81	0.83	0.82	1																
mFink1_6	0.76	0.78	0.8	0.81	0.84	1															
mFink1_7	0.78	0.79	0.8	0.82	0.81	0.82	1														
mFink1_8	0.75	0.76	0.78	0.76	0.79	0.81	0.77	1													
mFink1_9	0.75	0.78	0.8	0.78	0.83	0.82	0.81	0.87	1												
mFink1_10	0.73	0.71	0.72	0.79	0.8	0.79	0.79	0.77	0.79	1											
mFink2_1	0.51	0.51	0.52	0.58	0.57	0.58	0.56	0.52	0.58	0.63	1										
mFink2_2	0.5	0.47	0.5	0.55	0.56	0.56	0.53	0.52	0.54	0.62	0.91	1									
mFink2_3	0.48	0.45	0.48	0.56	0.51	0.52	0.52	0.5	0.51	0.61	0.88	0.91	1								
mFink2_4	0.45	0.41	0.44	0.52	0.47	0.49	0.47	0.46	0.49	0.56	0.85	0.89	0.9	1							
mFink2_5	0.43	0.38	0.43	0.47	0.45	0.5	0.47	0.47	0.48	0.57	0.83	0.86	0.88	0.91	1						
mKasper1	0.31	0.24	0.28	0.26	0.3	0.35	0.32	0.32	0.32	0.33	0.45	0.5	0.51	0.54	0.53	1					
mKasper2	0.33	0.27	0.32	0.28	0.31	0.31	0.32	0.33	0.3	0.34	0.45	0.52	0.54	0.55	0.53	0.92	1				
mKasper3	0.33	0.28	0.29	0.31	0.31	0.34	0.31	0.35	0.33	0.34	0.46	0.51	0.53	0.54	0.52	0.92	0.93	1			
mKasper4	0.29	0.26	0.29	0.29	0.29	0.32	0.31	0.33	0.31	0.33	0.48	0.52	0.54	0.57	0.55	0.93	0.94	0.96	1		
mKasper5	0.28	0.25	0.27	0.29	0.31	0.31	0.29	0.32	0.3	0.32	0.46	0.49	0.52	0.53	0.54	0.9	0.9	0.92	0.93	1	
mKleinb	0.37	0.37	0.29	0.3	0.34	0.3	0.35	0.38	0.36	0.39	0.39	0.4	0.42	0.44	0.45	0.49	0.48	0.52	0.52	0.51	1
moverall	0.61	0.59	0.6	0.64	0.64	0.65	0.63	0.63	0.65	0.67	0.79	0.81	0.81	0.82	0.82	0.73	0.74	0.76	0.76	0.74	0.58
